# Mid-Gestational Enlargement of Fetal Thalami in Women Exposed to Methadone during Pregnancy

**DOI:** 10.3389/fsurg.2014.00028

**Published:** 2014-07-21

**Authors:** Meredith Schulson, Anthony Liu, Tracey Björkman, Ann Quinton, Kristy P. Mann, Ron Benzie, Michael Peek, Ralph Nanan

**Affiliations:** ^1^Discipline of Paediatrics, Sydney Medical School – Nepean, The University of Sydney, Penrith, NSW, Australia; ^2^Charles Perkins Centre – Nepean, The University of Sydney, Penrith, NSW, Australia; ^3^Perinatal Research Centre, UQ Centre for Clinical Research, The University of Queensland, Herston, QLD, Australia; ^4^Discipline of Obstetrics and Gynaecology, Sydney Medical School – Nepean, The University of Sydney, Penrith, NSW, Australia; ^5^NHMRC Clinical Trials Centre, The University of Sydney, Camperdown, NSW, Australia

**Keywords:** pre-natal drug exposure, methadone, opiates, neurodevelopment, neuroimaging

## Abstract

Methadone maintenance therapy is the standard of care in many countries for opioid-dependent women who become pregnant. Despite recent evidence showing significant neurodevelopmental changes in children and adults exposed to both licit and illicit substances *in utero*, data on the effects of opioids in particular remains scarce. The purpose of this study was to examine the effects of opiate use, in particular methadone, on various fetal cortical and biometric growth parameters *in utero* using ultrasound measurements done at 18–22 weeks gestation. Head circumference (HC), bi-parietal diameter, lateral ventricle diameter, transcerebellar diameter, thalamic diameter, cisterna magna diameter, and femur length were compared between fetuses born to methadone-maintained mothers and non-substance using controls. A significantly larger thalamic diameter (0.05 cm, *p* = 0.01) was observed in the opiate-exposed group. Thalamic diameter/HC ratio was also significantly raised (0.03 mm, *p* = 0.01). We hypothesize here that the increase in thalamic diameter in opiate-exposed fetuses could potentially be explained by regional differences in opioid and serotonin receptor densities, an alteration in monoamine neurotransmitter systems, and an enhancement of the normal growth increase that occurs in the thalamus during mid-gestation.

## Introduction

Methadone maintenance therapy is the standard of care in many countries for opioid-dependent women who become pregnant (1, 2). Although there are many benefits to the use of methadone during pregnancy, it readily crosses the placenta, enters the bloodstream of the fetus and imposes potential risks (3, 4). The main documented adverse outcome of methadone use during pregnancy is the development of neonatal abstinence syndrome (NAS), with withdrawal being reported in more than 50% of infants born to methadone-maintained mothers (5). Opiate-exposed neonates have also been reported to be born prematurely and have lower birth weights, lengths, and head circumferences (HC) up to 5 years of age (6–8). Recent evidence shows that exposure to both licit and illicit substances causes significant structural brain changes in children, adolescents, and adults, which leads to the hypothesis that there may be significant effects on the brains of fetuses as well (9–11). These changes are diverse not only in terms of regions of the brain affected, but also in terms of the specific effects themselves, and occur on a cellular, macroscopic structural, and functional/behavioral level (9–13).

The size of a multitude of cortical and sub-cortical structures, the integrity of white matter tracts, changes in blood-flow pattern, and alterations in neurotransmitter levels have all been shown to be affected by different substances (9, 11, 12, 14). However, the data on the specific effects of opiates (methadone in particular) is scarce and only a few studies on their effects on brain development in humans can be identified (11, 15). One such study showed a reduction in the volumes of structures including the cerebral cortex, amygdala, and basal ganglia in children exposed primarily to heroin *in utero* (11). In an older study conducted with ultrasound, the thalamic cross-sectional area and HC were found to be larger in methadone-exposed infants at 6 months of age when compared to controls (15). Interestingly, in the rat opioids appear to selectively accumulate in the nervous tissues of fetal and pre-weaning rats, perhaps due to an increased permeability of their blood–brain barriers (16–18). Furthermore, research has shown that the limbic system, thalamus, and striatum appear to have among the highest concentration of opioid receptors (18), and therefore, it would seem likely that *in utero* opioid exposure would have the most significant effect in those areas. The studies mentioned above (11, 15) show some support for this notion, however, further evidence is needed. No evidence can be found specifically on the effect of opioid exposure in the human brain prenatally. Therefore, the purpose of this study was to examine the effects of opiate use, in particular methadone, on various fetal cortical and biometric growth parameters *in utero* using ultrasound measurements done at 18–22 weeks gestation. Use of such non-invasive and readily available measures could potentially allow for easier identification of the neurodevelopmental effects of opioid use during pregnancy.

## Materials and Methods

### Subjects

A retrospective medical record review was conducted on fetuses born to methadone-maintained pregnant women at a tertiary birthing unit between 2000 and 2006 inclusive. Women who had undergone their fetal anomaly scans (FAS) between gestational ages 18 and 22 weeks were included in our cohort. One hundred and eight control subjects who did not use illicit drugs or alcohol were identified from the local obstetric database and matched to our subjects based on maternal age, gender of the fetus, gestational age at FAS ± 5 days, and birth month ± 2 months. Exclusion criteria for both our subjects and controls included women with preeclampsia, hypertension, diabetes mellitus, multiple pregnancies, fetuses with congenital malformations, or non-satisfactory scans (that is unable to identify or measure required parameters). This study was approved by the Human Research Ethics Committees of both the Nepean Blue Mountains Local Health District and the University of Sydney.

### Parameters measured

Images were captured on the ultrasound machines GE Voluson 730 (GE Medical Systems, Piscataway, NJ, USA), GE Voluson i (GE Medical Systems, Piscataway, NJ, USA), or Medison Accuvix V20 Prestige (Samsung Medison, Seoul, South Korea). The measurements used for our data were taken by accredited perinatal sonographers. These included bi-parietal diameter (BPD), HC, femur length (FL), transcerebellar diameter (TCD), and lateral ventricle diameter. Additionally, thalamic diameter and cisterna magna diameter were measured using Adobe Photoshop v.7.0 (Adobe Systems Inc., USA). Gestational age of the fetus was based on last menstrual period (LMP) and confirmed by an ultrasound performed before 20 weeks gestation.

### Statistical analysis

Statistical analysis was performed using SPSS v.21.0 (SPSS Inc., Chicago, IL, USA). Descriptive statistics are reported as means and standard deviations. The data was confirmed to be normally distributed and variables were compared between the opiate-dependent group and the control group using independent samples *t*-tests. Adjusted analysis was undertaken using multiple regression modeling. A two tailed *p*-value of <0.05 was considered statistically significant.

## Results

Forty-two fetuses with FAS between 18 and 22 weeks were identified from our cohort of methadone-maintained mothers. The exact amount of opiate and other illicit drug use during pregnancy is intrinsically difficult to identify and is mainly dependent on self-reporting. However, our subjects were primarily opiate-dependent and ultimately maintained on methadone substitution therapy during their pregnancy. Although alcohol use was not considered an exclusion criterion, only 1 of 42 subjects reported using alcohol. Further details of this cohort are described in previous publications (19, 20) and other pertinent maternal and neonatal demographic characteristics are shown in Table 1.

**Table 1:** **Maternal and neonatal characteristics**.

	Opiate-exposed group, *n* = 42	Control group, *n* = 108
**Maternal characteristics**		
Age (years)	27.4 ± 4.3	27.4 ± 4.3
Smokers	39 (93%)	15 (14%)
Alcohol use	1 (2%)	0 (0%)
**Neonatal characteristics**		
Gender	20 female (48%), 22 male (52%)	53 female (49%), 55 male (51%)
Gestational age at FAS (weeks + days)	19 + 3 ± 5 days	19 + 3 ± 5 days
NAS treated with morphine	36 (86%)	0 (0%)
Urine drug screen at birth	37 (88%)	0 (0%)
Substances reported in UDS		
Methadone	35 (94%)	
Opiates	6 (16%)	
Benzodiazepines	4 (11%)	
Cannabis	10 (27%)	
Amphetamines	1 (3%)	

*NAS, neonatal abstinence syndrome; UDS, urinary drug screen; FAS, fetal anomaly scan*.

All parameters measured during the FAS were compared between the opiate – exposed group and the control group (Table 2). Of these, there were no statistically significant differences observed between the groups except for thalamic diameter. The diameter of the thalamus was, on average, 0.05 cm larger in the opiate-exposed group compared to the control group (*p* = 0.01). Adjusted for gestational age, the thalamic diameter was still significantly larger by 0.05 cm (95% CI: 0.10–0.85, *p* = 0.01) in the exposure group. The ratio of thalamic diameter/HC was also compared between the two groups and found to be significantly larger in the opiate-exposed group (0.90 ± 0.1 versus 0.87 ± 0.1 mm; *p* = 0.01). Finally, because of the known effect that cigarette smoking has on growth in the developing fetus (9, 19), comparisons of all parameters measured during the FAS between smokers and non-smokers were also performed (Table 3).

**Table 2:** **Comparison of FAS measurements between opiate – exposed fetuses and controls**.

Variable	Controls, *n* = 108 (cm ± SD)	Opiate-exposed, *n* = 42 (cm ± SD)	Difference (control-methadone) and 95% CI	*p*-Value
Bi-parietal diameter	4.44 (0.26)	4.43 (0.27)	0.01 (−0.08 to 0.11)	0.78
Head circumference	16.54 (0.96)	16.55 (1.04)	−0.01 (−0.36 to 0.34)	0.94
Lateral ventricle diameter	0.77 (0.13)	0.74 (0.09)	0.03 (−0.01 to 0.07)	0.19
Cerebellar diameter	1.9 (0.15)	1.92 (0.12)	−0.02 (−0.07 to 0.03)	0.46
Thalamus diameter	1.43 (0.10)	1.48 (0.10)	−0.05 (−0.09 to −0.01)	**0.01**
Cisterna magna diameter	0.40 (0.08)	0.42 (0.08)	−0.02 (−0.04 to 0.01)	0.32
Femur length	3.02 (0.24)	3.01 (0.28)	0 (−0.08 to 0.10)	0.85

*Bold font indicates significant difference*.

**Table 3:** **Comparison of FAS measurements between smokers and non-smokers**.

Variable	Non-smokers, *n* = 96 (cm ± SD)	Smokers, *n* = 54 (cm ± SD)	Difference (non-smokers – smokers) and 95% CI	*p*-Value
Bi-parietal diameter	4.45 (0.26)	4.43 (0.28)	0.02 (−0.07 to 0.11)	0.65
Head circumference	16.55 (0.92)	16.52 (1.08)	0.04 (−0.29 to 0.36)	0.83
Lateral ventricle diameter	0.76 (0.07)	0.76 (0.18)	0.00 (−0.04 to 0.04)	0.90
Cerebellar diameter	1.92 (0.11)	1.90 (0.19)	0.02 (−0.03 to 0.06)	0.49
Thalamus diameter	1.43 (0.10)	1.48 (0.10)	−0.04 (−0.08 to −0.01)	**0.02**
Cisterna magna diameter	0.40 (0.08)	0.41 (0.08)	−0.01 (−0.04 to 0.02)	0.53
Femur length	3.02 (0.23)	3.02 (0.29)	0 (−0.09 to 0.08)	0.89

*Bold font indicates significant difference*.

There was no statistically significant difference in any of the variables except thalamic diameter. The thalamus was, on average, 0.04 cm larger in the smoking group compared to the non-smoking group (*p* = 0.02). When comparing the thalamic diameter/HC ratio in the smokers versus non-smokers, the ratio was found to be significantly larger in the smoking group (0.90 ± 0.07 mm in smokers versus 0.86 ± 0.07 mm in non-smokers; *p* = 0.01). Smoking could not be controlled for in our patient and control group selection. As such, smoking was almost completely confounded by methadone use and it was not possible to model an interaction in this patient population. Table 4 provides a descriptive analysis, however, showing a trend toward a larger thalamic diameter with exposure to either cigarette smoking or opiate-exposure independently, with a possible cumulative effect of exposure to both.

**Table 4:** **Descriptive analysis of thalamic diameter in four exposure groups: non-smoking controls, non-smoking opiate-exposed, smoking controls, and smoking opiate-exposed**.

Group	Thalamus size (cm) Mean ± SD	95% CI
Non-smoking controls (*n* = 93)	1.43 ± 0.01	1.41–1.45
Non-smoking opiate-exposed (*n* = 3)	1.46 ± 0.05	1.35–1.56
Smoking controls (*n* = 15)	1.45 ± 0.03	1.39–1.50
Smoking opiate-exposed (*n* = 39)	1.48 ± 0.17	1.45–1.52

## Discussion

An analysis of several parameters measured during the FAS of opiate-exposed and control fetuses have demonstrated a significant increase in the thalamic diameter in opiate-exposed fetuses. The thalamic diameter/HC ratio was also significantly increased in the opiate-exposed group, indicating that the thalamus grew out of proportion with the rest of the head. This result was unexpected, and is difficult to explain due to limited literature on the topic. Only one previous study has looked specifically at thalamic size in neonates born to methadone-maintained mothers (15). This study used ultrasound to demonstrate that thalamic area was not significantly different at birth and at 1 month (though trended toward being smaller) when compared to controls. Between 1 and 6 months of age, however, the growth rate of the thalamus more than doubled that of the controls, resulting in significantly larger thalami at 6 months. Other neuroimaging studies using MRI have shown a decrease in thalamic volume after pre-natal exposure to alcohol (21), methamphetamine (22), and cocaine (9). However, these studies have all been performed on children and adolescents years after exposure to such substances. The pre-natal increase in thalamic size, we have demonstrated may potentially be explained by normal developmental patterns of the thalamus and also by regional differences in opiate-receptor density and monoamine neurotransmitter systems within the brain.

Overall, very little is known about the normal pre-natal development of the thalami. Both a recent 3D ultrasound report (23) and an older histochemical study (24), however, have demonstrated that growth of the thalamus does not progress linearly in humans, and that there is a striking increase in size from 20 to 28 weeks gestation, with slower growth both before and after that time. Interestingly, the thalamus has also been shown to have one of the highest concentrations of opioid receptors in the brain overall, with the density of those receptors increasing with gestational age and into adulthood (18, 25). Furthermore, the thalamus is reported to have one of the highest concentrations of serotonin transporters in the brain (26), and methadone exposure has been shown to increase the concentration of the serotonin metabolite 5-hydroxyindoleacetic acid (5-HIAA) particularly in the striatum, an area of the brain intimately connected to the thalamus (27). This is interesting, as many of the proposed neurodevelopmental effects of other substances such as methamphetamine, cocaine, and tobacco are attributed to alterations in monoamine neurotransmitter systems and these neurotransmitters reportedly play a key trophic role in brain development, particularly in the development of the thalamus (9, 28, 29). Therefore, our proposed hypothesis is that pre-natal opiate-exposure alters monoamine neurotransmitter systems and somehow “ramps-up” the normal developmental increase in thalamus size that occurs around mid-gestation; the thalamus being particularly susceptible due to the increased density of both opioid and serotonin receptors. The actual functional implications of such a change in thalamic size are unknown. However, as the thalamus is responsible for an array of critical functions such as relaying information between cortical areas and transmitting peripheral stimuli to the cortex (23), the changes we have demonstrated may well prove to be clinically significant.

Briefly, although it is difficult in this case to disentangle the effects of opiates versus cigarette smoking on the size of the thalamus in our subjects, it appears that nicotine may be independently causing an increase in thalamus size as well. If this is the case, it could potentially be due to nicotine’s ability to bind nicotinic acetylcholine receptors (nAChRs) and the fact that acetylcholine (ACh) plays a vital role in brain development and maturation. By binding nAChRs, nicotine has the ability to disrupt brain development and potentially alter the size of distinct brain regions (30, 31). Furthermore, nicotine, like methadone, alters monoamine systems and also has the potential to affect thalamic size in this manner (32). More research needs to be done however, specifically examining the effects of cigarette smoking on neurodevelopment.

Limitations of this research include the lack of post-natal follow up measurements as well as the inability to independently analyze the effects of opiates and nicotine on thalamic diameter. This study would best be performed longitudinally in the future, with smoking status incorporated as a controlled parameter, tracking changes in the thalamus diameter at birth and into childhood. Also, as previously stated, determining the exact amount of opiate and other illicit drug use during pregnancy in this cohort is intrinsically difficult to identify. Therefore, confounding factors such as the effect of other substances potentially used during pregnancy must be considered.

## Conclusion

Opiate-exposure (primarily methadone) is associated with a significant increase in thalamic diameter when compared to controls. This could potentially be attributed to regional differences in opioid and serotonin receptor densities, an alteration in monoamine neurotransmitter systems, and an enhancement of the normal growth increase that occurs in the thalamus during mid-gestation. Pre-natal nicotine exposure also appears to increase the size of the thalamus, though the effects are difficult to separate from that of the opiates. None of the other growth parameters measured during the FAS between 18 and 22 weeks were significantly different to controls.

## Conflict of Interest Statement

The authors declare that the research was conducted in the absence of any commercial or financial relationships that could be construed as a potential conflict of interest.

## References

[1] Dunlop C Panjari M O’Sulllivan H Henschke P Love V Ritter A Clinical Guidelines for the Use of Buprenorphine in Pregnancy. In: Victoria DHS, editor. Fitzroy: Turning Point Alcohol and Drug Centre (2003). p. 1–49.

[2] Wong S Ordean A Kahan M. Maternal fetal medicine committee, family physicians advisory committee, medico-legal committee, society of obstetricians and gynaecologists of Canada. Substance use in pregnancy. J Obstetr Gynaecol Can (2011) 33:367–84. 21501542 10.1016/S1701-2163(16)34855-1

[3] Burns L Mattick RP Lim K Wallace C. Methadone in pregnancy: treatment retention and neonatal outcomes. Addiction (2007) 102:264–70. 10.1111/j.1360-0443.2006.01651.x 17222281

[4] Nekhayeva IA Nanovskaya TN Deshmukh SV Zharikova OL Hankins GDV Ahmed MS. Bidirectional transfer of methadone across the human placenta. Biochem Pharmacol (2005) 69:187–97. 10.1016/j.bcp.2004.09.008 15588727

[5] Jones HE Finnegan LP Kaltenbach K. Methadone and buprenorphine for the management of opioid dependence in pregnancy. Drugs (2012) 72:747–57. 10.2165/11632820-000000000-00000 22512363

[6] Brown HL Britton KA Mahaffey D Brizendine E Hiett AK Turnquest MA. Methadone maintenance in pregnancy: a reappraisal. Am J Obstet Gynecol (1998) 179:459–63. 10.1016/S0002-9378(98)70379-5 9731853

[7] Kandall SR Albin S Lowinson J Berle B Eidelman AI Gartner LM. Differential effects of maternal heroin and methadone use on birth weight. Pediatrics (1976) 58:681–5. 980601

[8] Van Baar A de Graaff BM. Cognitive development at preschool-age of infants of drug-dependent mothers. Dev Med Child Neurol (1994) 36:1063–75. 10.1111/j.1469-8749.1994.tb11809.x 7958521

[9] Derauf C Kekatpure M Neyzi N Lester B Kosofsky B. Neuroimaging of children following prenatal drug exposure. Sem Cell Dev Biol (2009) 20:441–54. 10.1016/j.semcdb.2009.03.001 19560049 PMC2704485

[10] Roussotte F Soderberg L Sowell E. Structural, metabolic, and functional brain abnormalities as a result of prenatal exposure to drugs of abuse: evidence from neuroimaging. Neuropsychol Rev (2010) 20:376–97. 10.1007/s11065-010-9150-x 20978945 PMC2988996

[11] Walhovd KB Moe V Slinning K Due-Tønnessen P Bjørnerud A Dale AM Volumetric cerebral characteristics of children exposed to opiates and other substances in utero. Neuroimage (2007) 36:1331–44. 10.1016/j.neuroimage.2007.03.070 17513131 PMC2039875

[12] Rao H Wang J Giannetta J Korczykowski M Shera D Avants B Altered resting cerebral blood flow in adolescents with in utero cocaine exposure revealed by perfusion functional MRI. Pediatrics (2007) 120:1245–54. 10.1542/peds.2006-2596 17974718

[13] Yanai J Huleihel R Izrael M Metsuyanim S Shahak H Vatury O Functional changes after prenatal opiate exposure related to opiate receptors’ regulated alterations in cholinergic innervation. Int J Neuropsychopharmacol (2003) 6:253–65. 10.1017/S1461145703003523 12974992

[14] Lebel C Rasmussen C Wyper K Walker L Andrew G Yager J Brain diffusion abnormalities in children with fetal alcohol spectrum disorder. Alcohol Clin Exp Res (2008) 32:1732–40. 10.1111/j.1530-0277.2008.00750.x 18671811

[15] Pasto ME Deiiing J Graziani LJ Ehrlich S Finnegan LP Disparity in hemispheric and thalamic growth in infants undergoing abstinence. NIDA Res Monogr (1986) 67:342–8. 3092088

[16] Peters MA Turnbow M Buchenauer D The distribution of methadone in the nonpregnant, pregnant, and fetal rat after acute methadone treatment. J Pharmacol Exp Ther (1972) 181:273–8. 5030672

[17] Shah NS Donald AG Pharmacological effects and metabolic fate of levo-methadone during postnatal development in rat. J Pharmacol Exp Ther (1979) 208:491–7. 430365

[18] Vathy I. Prenatal opiate exposure: long term CNS consequences in the stress system of the offspring. Psychoneuroendocrinology (2002) 27:273–83. 10.1016/S0306-4530(01)00049-X 11750783

[19] Delsing C Van Den Wittendoer E Liu AJW Peek MJ Quinton A Mongelli M The relationship between maternal opiate use, amphetamine use, and smoking on fetal growth. Aust N Zeal J Obstetr Gynaecol (2011) 51:446–51. 10.1111/j.1479-828X.2011.01342.x 21806595

[20] Liu AJW Jones MP Murray H Cook C Nanan R. Perinatal risk factors for the neonatal abstinence syndrome in infants born to women on methadone maintenance therapy. Aust N Zeal J Obstetr Gynaecol (2010) 50:253–8. 10.1111/j.1479-828X.2010.01168.x 20618243

[21] Mattson SN Riley EP Sowell ER Jernigan TL Sobel DF Jones KL. A decrease in the size of the basal ganglia in children with fetal alcohol syndrome. Alcohol Clin Exp Res (1996) 20:1088–93. 10.1111/j.1530-0277.1996.tb01951.x 8892532

[22] Sowell ER Leow AD Bookheimer SY Smith LM O’Connor MJ Kan E Differentiating prenatal exposure to methamphetamine and alcohol versus alcohol and not methamphetamine using tensor-based brain morphometry and discriminant analysis. J Neurosci (2010) 30:3876–85. 10.1523/JNEUROSCI.4967-09.2010 20237258 PMC2847574

[23] Sotiriadis A Dimitrakopoulos I Eleftheriade M Agorastos T Makrydimas G. Thalamic volume measurement in normal fetuses using three-dimensional sonography. Journal of Clinical Ultrasound (2012) 40:207–13. 10.1002/jcu.21888 22286969

[24] Kostovic I Goldman-Rakic PS. Transient cholinesterase staining in the mediodorsal nucleus of the thalamus and its connections in the developing human and monkey brain. J Comp Neurol (1983) 219:431–47. 10.1002/cne.902190405 6196382

[25] Villiger JW Taylor KM Gluckman PD. Ontogenesis of opiate receptors in regions of the ovine brain. Pediatr Pharmacol (1982) 2:349–56. 6099887

[26] Frankle WG Huang Y Hwang DR. Comparative evaluation of serotonin transporter radioligands 11C-DASB and 11C-McN 5652 in healthy humans. J Nuclear Med (2004) 45:682–94. 15073266

[27] Robinson SE Maher JR Wallace MJ Kunko PM. Perinatal methadone exposure affects dopamine, norepinephrine, and serotonin in the weanling rat. Neurotoxicol Teratol (1997) 19:295–303. 10.1016/S0892-0362(97)00018-4 9253008

[28] Esaki T Cook M Shimoji K Murphy DL Sokoloff L Holmes A. Developmental disruption of serotonin transporter function impairs cerebral responses to whisker stimulation in mice. Proc Natl Acad Sci U S A (2005) 102:5582–7. 10.1073/pnas.0501509102 15809439 PMC556265

[29] Monckton JE McCormick DA. Neuromodulatory role of serotonin in the ferret thalamus. J Neurophysiol (2002) 87:2124–36. 10.1152/jn.00650.2001 11929930

[30] Slotkin TA. Cholinergic systems in brain development and disruption by neurotoxicants: nicotine, environmental tobacco smoke, organophosphates. Toxicol Appl Pharmacol (2004) 198:132–51. 10.1016/j.taap.2003.06.001 15236950

[31] Slotkin TA Seidler FJ Qiao D Aldridge JE Tate CA Cousins MM Effects of prenatal nicotine exposure on primate brain development and attempted amelioration with supplemental choline or vitamin C: neurotransmitter receptors, cell signaling and cell development biomarkers in fetal brain regions of rhesus monkeys. Neuropsychopharmacology (2005) 30:129–44. 10.1038/sj.npp.1300544 15316571

[32] Muneoka K Ogawa T Kamei K Muraoka S Tomiyoshi R Mumura Y Prenatal nicotine exposure affects the development of the central serotonergic system as well as the dopaminergic system in rat offspring: involvement of route of drug administration. Brain Res Dev Brain Res (1997) 102:117–26. 10.1016/S0165-3806(97)00092-8 9298240

